# (*S*)-1-(2-Chloro­phen­yl)-2-oxocyclo­hexan-1-aminium d-tartrate

**DOI:** 10.1107/S1600536811006131

**Published:** 2011-02-26

**Authors:** Marhaba Hojahmat, Guangrong Zheng, Max Siegler, Sean Parkin, Manfred Biermann, Peter A. Crooks

**Affiliations:** a Yaupon Therapeutics, Inc., 259 North Radnor-Chester Road, Suite 205, Radnor, PA 19087, USA; b Department of Pharmaceutical Sciences, College of Pharmacy, University of Kentucky, Lexington, KY 40536, USA; c Department of Chemistry, University of Kentucky, Lexington, KY 40536, USA; d Resodyn Corporation, 130 North Main Street, Suite 600, Butte, MT 59701, USA

## Abstract

In the title compound, C_12_H_15_ClNO^+^·C_4_H_5_O_6_^−^, the cyclo­hexa­none ring adopts a chair conformation. The benzene ring is significantly twisted so that it is in an almost perpendicular position to the C—N bond with a C_Ar_—C_Ar_—C—N torsion angle of −96.5 (5)°. Intermolecular N—H⋯O and O—H⋯O hydrogen bonds are observed in the crystal structure.

## Related literature

For background to ketamine, see: Holtman (2006 ▶); Holtman *et al.* (2006 ▶); Heshmati *et al.* (2003 ▶); Kohrs & Durieux (1998 ▶). For the synthesis, see: Hong & Davisson (1982 ▶); Parcell & Sanchez (1981 ▶). 
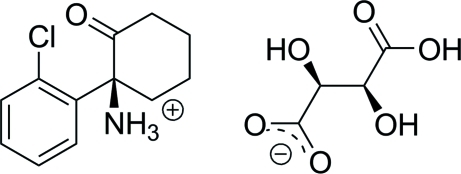


## Experimental

### 

#### Crystal data

C_12_H_15_ClNO^+^·C_4_H_5_O_6_^−^
*M* *_r_* = 373.78
Orthorhombic, 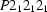

*a* = 7.1411 (2) Å
*b* = 9.9878 (4) Å
*c* = 23.7530 (11) Å
*V* = 1694.16 (11) Å^3^
*Z* = 4
Mo *K*α radiation
μ = 0.27 mm^−1^
*T* = 90 K
0.20 × 0.20 × 0.03 mm

#### Data collection

Nonius KappaCCD diffractometer
Absorption correction: multi-scan (*SCALEPACK*; Otwinowski & Minor, 1997 ▶) *T*_min_ = 0.949, *T*_max_ = 0.992
13735 measured reflections
2986 independent reflections
1519 reflections with *I* > 2σ(*I*)
*R*_int_ = 0.110

#### Refinement

*R*[*F* ^2^ > 2σ(*F* ^2^)] = 0.060
*wR*(*F* ^2^) = 0.139
*S* = 0.96
2986 reflections
230 parameters
H-atom parameters constrained
Δρ_max_ = 0.28 e Å^−3^
Δρ_min_ = −0.27 e Å^−3^
Absolute structure: Flack (1983 ▶), 1241 Friedel pairs
Flack parameter: 0.10 (10)

### 

Data collection: *COLLECT* (Nonius, 1998 ▶); cell refinement: *SCALEPACK* (Otwinowski & Minor, 1997 ▶); data reduction: *DENZO-SMN* (Otwinowski & Minor, 1997 ▶); program(s) used to solve structure: *SHELXS97* (Sheldrick, 2008 ▶); program(s) used to refine structure: *SHELXL97* (Sheldrick, 2008 ▶); molecular graphics: *XP* in *SHELXTL* (Sheldrick, 2008 ▶); software used to prepare material for publication: *SHELXL97* and local procedures.

## Supplementary Material

Crystal structure: contains datablocks global, I. DOI: 10.1107/S1600536811006131/hg2766sup1.cif

Structure factors: contains datablocks I. DOI: 10.1107/S1600536811006131/hg2766Isup2.hkl

Additional supplementary materials:  crystallographic information; 3D view; checkCIF report

## Figures and Tables

**Table 1:** Hydrogen-bond geometry (Å, °)

*D*—H⋯*A*	*D*—H	H⋯*A*	*D*⋯*A*	*D*—H⋯*A*
N1—H1*A*⋯O7^i^	0.91	1.81	2.715 (5)	176
N1—H1*B*⋯O4	0.91	2.05	2.856 (5)	147
N1—H1*C*⋯O3^ii^	0.91	2.29	2.893 (5)	123
N1—H1*C*⋯O5^ii^	0.91	2.37	3.001 (5)	126
O2—H2*A*⋯O1^iii^	0.84	2.60	3.388 (5)	157
O5—H5*A*⋯O6^iv^	0.84	2.09	2.864 (5)	153
O6—H6⋯O4^v^	0.84	1.64	2.460 (5)	166
O6—H6⋯O3^v^	0.84	2.62	3.265 (5)	134

Symmetry codes: (i) 
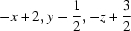
; (ii) 
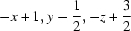
; (iii) 
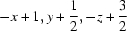
; (iv) 
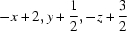
; (v) 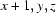.

**Fig. 1.**
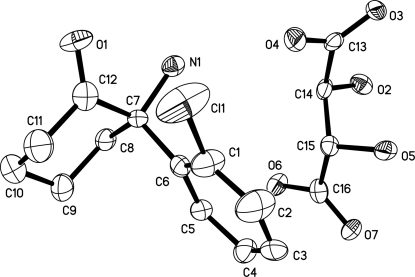
A view of the molecules with the atom numbering scheme. Displacement ellipsoids are drawn at the 50% probability level.

## supplementary crystallographic information

### Comment

Ketalar^TM^, the racemic mixture of *R-* and *S*-Ketamines is becoming the sedative and anesthetic of choice for emergency sedation in children and victims with unknown medical history, *e.g.* from traffic accidents to battlefield conditions, because it causes minimal respiratory depression in comparison to other anesthetics (Heshmati *et al.*, 2003). *S*-Ketamine was found 3–4 times more potent as an anesthetic than its *R*-enantiomer, and twice as potent as Ketalar^TM^ with fewer side effects such as psychedelic, disorientation and anxiety (Kohrs & Durieux, 1998). *S*-Norketamine, the major metabolite of *S*-Ketamine in humans and animals, is emerging as a novel drug for treatment of neuropathic pain (Holtman *et al.*, 2006) and for analgesia (Holtman, 2006). To confirm the absolute configuration of (+)-norketamine, herein we report on the X-ray crystallographic characterization of crystalline *S*-norketamine D-tartrate salt.

### Experimental

*S*-Norketamine was obtained as a D-tartrate salt form *via* chiral resolution of racemic norketamine by fractional crystallization of the D-tartrate salt (Hong & Davisson, 1982). Racemic norketamine was produced in large quantity according to literature report (Parcell & Sanchez, 1981). The chiral purity of the product was determined by chiral HPLC on a Chiralcel OJ—H column, and afforded ee% > 99%. The specific rotation of the tartrate salt is [*a*]_D_ + 55.7° (c = 2, H_2_O), and the specific rotations for the corresponding corresponding free base and HCl salt are [*a*]_D_ + 3.6° (c = 2, EtOH) and [*a*]_D_ + 75.9° (c = 1, H_2_O), respectively.

### Refinement

H atoms were found in difference Fourier maps and subsequently placed in idealized positions with constrained distances of 0.95 Å (C_Ar_H), 1.00 Å (*R*_3_CH), 0.99 Å (*R*_2_CH_2_), 0.84 Å (O—H), 0.91 Å (NH_3_), and with *U*_iso_(H) values set to either 1.2*U*_eq_ or 1.5*U*_eq_ (NH_3_, OH) of the attached atom.

### Crystal data

**Table:**

C_12_H_15_ClNO^+^·C_4_H_5_O_6_^−^	*F*(000) = 784
*M**_r_* = 373.78	*D*_x_ = 1.465 Mg m^−^^3^
Orthorhombic, *P*2_1_2_1_2_1_	Mo *K*α radiation, λ = 0.71073 Å
Hall symbol: P 2ac 2ab	Cell parameters from 2155 reflections
*a* = 7.1411 (2) Å	θ = 1.0–27.5°
*b* = 9.9878 (4) Å	µ = 0.27 mm^−^^1^
*c* = 23.7530 (11) Å	*T* = 90 K
*V* = 1694.16 (11) Å^3^	Plate, colourless
*Z* = 4	0.20 × 0.20 × 0.03 mm

### Data collection

**Table:**

Nonius KappaCCD diffractometer	2986 independent reflections
Radiation source: fine-focus sealed tube	1519 reflections with *I* > 2σ(*I*)
graphite	*R*_int_ = 0.110
Detector resolution: 9.1 pixels mm^-1^	θ_max_ = 25.0°, θ_min_ = 1.7°
ω scans at fixed χ = 55°	*h* = −8→8
Absorption correction: multi-scan (*SCALEPACK*; Otwinowski & Minor, 1997)	*k* = −11→11
*T*_min_ = 0.949, *T*_max_ = 0.992	*l* = −27→28
13735 measured reflections	

### Refinement

**Table:**

Refinement on *F*^2^	Secondary atom site location: difference Fourier map
Least-squares matrix: full	Hydrogen site location: inferred from neighbouring sites
*R*[*F*^2^ > 2σ(*F*^2^)] = 0.060	H-atom parameters constrained
*wR*(*F*^2^) = 0.139	*w* = 1/[σ^2^(*F*_o_^2^) + (0.0568*P*)^2^] where *P* = (*F*_o_^2^ + 2*F*_c_^2^)/3
*S* = 0.96	(Δ/σ)_max_ < 0.001
2986 reflections	Δρ_max_ = 0.28 e Å^−^^3^
230 parameters	Δρ_min_ = −0.27 e Å^−^^3^
0 restraints	Absolute structure: Flack (1983), 1241 Friedel pairs
Primary atom site location: structure-invariant direct methods	Flack parameter: 0.10 (10)

### Special details

**Table:**

Geometry. All e.s.d.'s (except the e.s.d. in the dihedral angle between two l.s. planes) are estimated using the full covariance matrix. The cell e.s.d.'s are taken into account individually in the estimation of e.s.d.'s in distances, angles and torsion angles; correlations between e.s.d.'s in cell parameters are only used when they are defined by crystal symmetry. An approximate (isotropic) treatment of cell e.s.d.'s is used for estimating e.s.d.'s involving l.s. planes.
Refinement. Refinement of *F*^2^ against ALL reflections. The weighted *R*-factor *wR* and goodness of fit *S* are based on *F*^2^, conventional *R*-factors *R* are based on *F*, with *F* set to zero for negative *F*^2^. The threshold expression of *F*^2^ > 2σ(*F*^2^) is used only for calculating *R*-factors(gt) *etc*. and is not relevant to the choice of reflections for refinement. *R*-factors based on *F*^2^ are statistically about twice as large as those based on *F*, and *R*- factors based on ALL data will be even larger.

### Fractional atomic coordinates and isotropic or equivalent isotropic displacement parameters (Å^2^)

**Table:**

	*x*	*y*	*z*	*U*_iso_*/*U*_eq_	
Cl1	0.3664 (2)	0.71723 (16)	0.91603 (9)	0.0776 (7)	
O1	0.4054 (5)	0.4009 (4)	0.92986 (16)	0.0391 (11)	
N1	0.5914 (5)	0.4734 (4)	0.83868 (16)	0.0254 (12)	
H1A	0.6786	0.4352	0.8158	0.038*	
H1B	0.5453	0.5485	0.8220	0.038*	
H1C	0.4964	0.4144	0.8448	0.038*	
C1	0.5939 (7)	0.7596 (6)	0.8988 (2)	0.0418 (17)	
C2	0.6335 (10)	0.8933 (6)	0.8922 (3)	0.057 (2)	
H2	0.5381	0.9585	0.8973	0.069*	
C3	0.8128 (10)	0.9322 (6)	0.8779 (2)	0.0449 (18)	
H3	0.8403	1.0242	0.8720	0.054*	
C4	0.9520 (9)	0.8374 (6)	0.8723 (2)	0.0339 (15)	
H4	1.0764	0.8643	0.8638	0.041*	
C5	0.9097 (7)	0.7021 (5)	0.8790 (2)	0.0282 (15)	
H5	1.0058	0.6375	0.8740	0.034*	
C6	0.7292 (7)	0.6595 (5)	0.8931 (2)	0.0246 (14)	
C7	0.6805 (7)	0.5100 (5)	0.8940 (2)	0.0226 (13)	
C8	0.8535 (7)	0.4174 (5)	0.9031 (2)	0.0291 (14)	
H8A	0.8155	0.3229	0.8978	0.035*	
H8B	0.9501	0.4387	0.8746	0.035*	
C9	0.9367 (8)	0.4348 (6)	0.9620 (2)	0.0399 (16)	
H9A	1.0470	0.3756	0.9662	0.048*	
H9B	0.9791	0.5285	0.9669	0.048*	
C10	0.7954 (9)	0.4015 (6)	1.0067 (2)	0.0489 (18)	
H10A	0.8503	0.4184	1.0443	0.059*	
H10B	0.7626	0.3054	1.0043	0.059*	
C11	0.6186 (9)	0.4861 (7)	0.9995 (2)	0.0505 (19)	
H11A	0.5231	0.4588	1.0274	0.061*	
H11B	0.6481	0.5819	1.0055	0.061*	
C12	0.5447 (9)	0.4655 (5)	0.9409 (2)	0.0321 (14)	
C13	0.5503 (8)	0.6596 (5)	0.6984 (3)	0.0266 (14)	
C14	0.7637 (7)	0.6537 (5)	0.6976 (2)	0.0242 (14)	
H14	0.8039	0.5662	0.7141	0.029*	
C15	0.8443 (7)	0.7649 (5)	0.7331 (2)	0.0218 (13)	
H15	0.7942	0.7538	0.7721	0.026*	
C16	1.0558 (8)	0.7639 (6)	0.7370 (2)	0.0255 (14)	
O2	0.8330 (5)	0.6619 (4)	0.64159 (14)	0.0297 (10)	
H2A	0.7541	0.7012	0.6212	0.045*	
O3	0.4634 (5)	0.6867 (3)	0.65555 (16)	0.0291 (10)	
O4	0.4745 (5)	0.6434 (3)	0.74860 (16)	0.0334 (10)	
O5	0.7747 (5)	0.8881 (3)	0.71191 (15)	0.0287 (10)	
H5A	0.8366	0.9517	0.7257	0.043*	
O6	1.1301 (5)	0.6502 (3)	0.74840 (16)	0.0304 (9)	
H6	1.2464	0.6547	0.7436	0.046*	
O7	1.1404 (5)	0.8705 (3)	0.72982 (14)	0.0269 (9)	

### Atomic displacement parameters (Å^2^)

**Table:**

	*U*^11^	*U*^22^	*U*^33^	*U*^12^	*U*^13^	*U*^23^
Cl1	0.0331 (10)	0.0394 (9)	0.160 (2)	0.0090 (9)	0.0157 (11)	−0.0109 (12)
O1	0.029 (3)	0.038 (2)	0.050 (3)	−0.0149 (19)	0.003 (2)	−0.008 (2)
N1	0.025 (3)	0.019 (2)	0.032 (3)	0.001 (2)	0.000 (2)	−0.002 (2)
C1	0.031 (4)	0.027 (4)	0.068 (5)	0.004 (3)	−0.004 (3)	−0.005 (3)
C2	0.045 (4)	0.030 (4)	0.096 (6)	0.009 (4)	−0.009 (4)	−0.008 (4)
C3	0.066 (5)	0.026 (4)	0.042 (4)	−0.011 (4)	−0.025 (4)	−0.004 (3)
C4	0.041 (4)	0.033 (4)	0.028 (3)	−0.009 (3)	0.003 (3)	0.003 (3)
C5	0.030 (4)	0.029 (4)	0.026 (3)	−0.006 (3)	−0.002 (3)	0.001 (3)
C6	0.031 (3)	0.026 (3)	0.017 (3)	−0.001 (3)	−0.001 (3)	0.000 (3)
C7	0.024 (3)	0.021 (3)	0.023 (3)	−0.005 (3)	−0.005 (3)	−0.001 (3)
C8	0.030 (3)	0.021 (3)	0.036 (4)	0.001 (3)	−0.005 (3)	0.005 (3)
C9	0.043 (4)	0.037 (4)	0.040 (4)	0.000 (3)	−0.004 (4)	0.010 (3)
C10	0.053 (5)	0.055 (5)	0.039 (4)	−0.008 (4)	−0.010 (4)	0.010 (4)
C11	0.056 (5)	0.068 (5)	0.027 (4)	−0.013 (4)	0.016 (4)	−0.004 (4)
C12	0.035 (4)	0.028 (4)	0.033 (4)	0.009 (3)	0.005 (3)	−0.001 (3)
C13	0.023 (3)	0.014 (3)	0.043 (4)	−0.003 (3)	−0.007 (3)	0.005 (3)
C14	0.023 (3)	0.022 (3)	0.028 (4)	−0.002 (3)	0.002 (3)	0.010 (3)
C15	0.018 (3)	0.023 (3)	0.025 (3)	0.003 (3)	0.001 (3)	0.003 (3)
C16	0.031 (4)	0.030 (4)	0.016 (3)	0.006 (3)	0.007 (3)	0.001 (3)
O2	0.030 (2)	0.031 (2)	0.028 (2)	0.004 (2)	−0.0047 (19)	−0.0057 (18)
O3	0.025 (2)	0.022 (2)	0.040 (2)	0.0016 (18)	−0.012 (2)	0.0039 (19)
O4	0.027 (2)	0.035 (2)	0.039 (2)	−0.0003 (19)	−0.005 (2)	0.010 (2)
O5	0.028 (2)	0.014 (2)	0.044 (2)	0.0049 (17)	−0.0106 (19)	−0.0062 (19)
O6	0.013 (2)	0.027 (2)	0.052 (3)	0.0024 (19)	0.002 (2)	0.010 (2)
O7	0.027 (2)	0.023 (2)	0.031 (2)	−0.0046 (19)	0.0049 (19)	0.0026 (18)

### Geometric parameters (Å, °)

**Table:**

Cl1—C1	1.728 (6)	C9—H9A	0.9900
O1—C12	1.214 (6)	C9—H9B	0.9900
N1—C7	1.505 (6)	C10—C11	1.529 (8)
N1—H1A	0.9100	C10—H10A	0.9900
N1—H1B	0.9100	C10—H10B	0.9900
N1—H1C	0.9100	C11—C12	1.503 (8)
C1—C2	1.374 (8)	C11—H11A	0.9900
C1—C6	1.397 (7)	C11—H11B	0.9900
C2—C3	1.380 (8)	C13—O3	1.223 (6)
C2—H2	0.9500	C13—O4	1.320 (6)
C3—C4	1.379 (7)	C13—C14	1.525 (7)
C3—H3	0.9500	C14—O2	1.423 (5)
C4—C5	1.393 (7)	C14—C15	1.508 (6)
C4—H4	0.9500	C14—H14	1.0000
C5—C6	1.398 (7)	C15—O5	1.419 (5)
C5—H5	0.9500	C15—C16	1.513 (6)
C6—C7	1.533 (7)	C15—H15	1.0000
C7—C12	1.542 (7)	C16—O7	1.237 (6)
C7—C8	1.558 (7)	C16—O6	1.282 (6)
C8—C9	1.531 (7)	O2—H2A	0.8400
C8—H8A	0.9900	O5—H5A	0.8400
C8—H8B	0.9900	O6—H6	0.8400
C9—C10	1.501 (7)		
			
C7—N1—H1A	109.5	C10—C9—H9B	109.4
C7—N1—H1B	109.5	C8—C9—H9B	109.4
H1A—N1—H1B	109.5	H9A—C9—H9B	108.0
C7—N1—H1C	109.5	C9—C10—C11	110.7 (5)
H1A—N1—H1C	109.5	C9—C10—H10A	109.5
H1B—N1—H1C	109.5	C11—C10—H10A	109.5
C2—C1—C6	122.9 (6)	C9—C10—H10B	109.5
C2—C1—Cl1	117.3 (5)	C11—C10—H10B	109.5
C6—C1—Cl1	119.8 (4)	H10A—C10—H10B	108.1
C1—C2—C3	119.5 (6)	C12—C11—C10	108.5 (5)
C1—C2—H2	120.2	C12—C11—H11A	110.0
C3—C2—H2	120.2	C10—C11—H11A	110.0
C4—C3—C2	119.9 (6)	C12—C11—H11B	110.0
C4—C3—H3	120.0	C10—C11—H11B	110.0
C2—C3—H3	120.0	H11A—C11—H11B	108.4
C3—C4—C5	119.9 (6)	O1—C12—C11	124.0 (6)
C3—C4—H4	120.1	O1—C12—C7	120.9 (5)
C5—C4—H4	120.1	C11—C12—C7	114.1 (5)
C4—C5—C6	121.5 (5)	O3—C13—O4	124.8 (5)
C4—C5—H5	119.2	O3—C13—C14	120.4 (5)
C6—C5—H5	119.2	O4—C13—C14	114.6 (5)
C1—C6—C5	116.3 (5)	O2—C14—C15	110.3 (4)
C1—C6—C7	122.6 (5)	O2—C14—C13	110.9 (4)
C5—C6—C7	120.6 (5)	C15—C14—C13	110.3 (5)
N1—C7—C6	108.6 (4)	O2—C14—H14	108.4
N1—C7—C12	107.1 (4)	C15—C14—H14	108.4
C6—C7—C12	115.7 (4)	C13—C14—H14	108.4
N1—C7—C8	108.2 (4)	O5—C15—C14	107.9 (4)
C6—C7—C8	113.6 (4)	O5—C15—C16	112.2 (4)
C12—C7—C8	103.2 (4)	C14—C15—C16	114.3 (4)
C9—C8—C7	111.5 (4)	O5—C15—H15	107.4
C9—C8—H8A	109.3	C14—C15—H15	107.4
C7—C8—H8A	109.3	C16—C15—H15	107.4
C9—C8—H8B	109.3	O7—C16—O6	126.1 (5)
C7—C8—H8B	109.3	O7—C16—C15	118.3 (5)
H8A—C8—H8B	108.0	O6—C16—C15	115.6 (5)
C10—C9—C8	111.1 (5)	C14—O2—H2A	109.5
C10—C9—H9A	109.4	C15—O5—H5A	109.5
C8—C9—H9A	109.4	C16—O6—H6	109.5
			
C6—C1—C2—C3	1.5 (10)	C9—C10—C11—C12	55.6 (7)
Cl1—C1—C2—C3	−179.4 (5)	C10—C11—C12—O1	107.0 (6)
C1—C2—C3—C4	−2.1 (10)	C10—C11—C12—C7	−61.8 (6)
C2—C3—C4—C5	2.2 (9)	N1—C7—C12—O1	6.8 (6)
C3—C4—C5—C6	−1.8 (8)	C6—C7—C12—O1	128.1 (5)
C2—C1—C6—C5	−1.0 (8)	C8—C7—C12—O1	−107.2 (5)
Cl1—C1—C6—C5	179.9 (4)	N1—C7—C12—C11	175.9 (5)
C2—C1—C6—C7	−173.0 (5)	C6—C7—C12—C11	−62.8 (6)
Cl1—C1—C6—C7	7.9 (7)	C8—C7—C12—C11	61.9 (6)
C4—C5—C6—C1	1.2 (8)	O3—C13—C14—O2	−9.2 (7)
C4—C5—C6—C7	173.3 (5)	O4—C13—C14—O2	175.6 (4)
C1—C6—C7—N1	75.1 (6)	O3—C13—C14—C15	113.3 (5)
C5—C6—C7—N1	−96.5 (5)	O4—C13—C14—C15	−61.9 (6)
C1—C6—C7—C12	−45.3 (7)	O2—C14—C15—O5	65.8 (5)
C5—C6—C7—C12	143.0 (5)	C13—C14—C15—O5	−57.0 (6)
C1—C6—C7—C8	−164.5 (5)	O2—C14—C15—C16	−59.7 (6)
C5—C6—C7—C8	23.9 (6)	C13—C14—C15—C16	177.5 (5)
N1—C7—C8—C9	−172.2 (4)	O5—C15—C16—O7	9.9 (7)
C6—C7—C8—C9	67.1 (6)	C14—C15—C16—O7	133.1 (5)
C12—C7—C8—C9	−59.0 (5)	O5—C15—C16—O6	−170.8 (4)
C7—C8—C9—C10	59.8 (6)	C14—C15—C16—O6	−47.6 (6)
C8—C9—C10—C11	−56.0 (7)		

### Hydrogen-bond geometry (Å, °)

**Table:**

*D*—H···*A*	*D*—H	H···*A*	*D*···*A*	*D*—H···*A*
N1—H1A···O7^i^	0.91	1.81	2.715 (5)	176
N1—H1B···O4	0.91	2.05	2.856 (5)	147
N1—H1C···O1	0.91	2.13	2.642 (5)	115
N1—H1C···O3^ii^	0.91	2.29	2.893 (5)	123
N1—H1C···O5^ii^	0.91	2.37	3.001 (5)	126
O2—H2A···O3	0.84	2.24	2.672 (5)	112
O2—H2A···O1^iii^	0.84	2.60	3.388 (5)	157
O5—H5A···O6^iv^	0.84	2.09	2.864 (5)	153
O6—H6···O4^v^	0.84	1.64	2.460 (5)	166
O6—H6···O3^v^	0.84	2.62	3.265 (5)	134

Symmetry codes: (i) −*x*+2, *y*−1/2, −*z*+3/2; (ii) −*x*+1, *y*−1/2, −*z*+3/2; (iii) −*x*+1, *y*+1/2, −*z*+3/2; (iv) −*x*+2, *y*+1/2, −*z*+3/2; (v) *x*+1, *y*, *z*.
